# Silencing of MUC20 suppresses the malignant character of pancreatic ductal adenocarcinoma cells through inhibition of the HGF/MET pathway

**DOI:** 10.1038/s41388-018-0403-0

**Published:** 2018-07-11

**Authors:** Syue-Ting Chen, Ting-Chun Kuo, Ying-Yu Liao, Mei-Chun Lin, Yu-Wen Tien, Min-Chuan Huang

**Affiliations:** 1 Graduate Institute of Anatomy and Cell Biology, College of Medicine, National Taiwan University, Taipei, Taiwan; 2 Department of Surgery, National Taiwan University Hospital, Taipei, Taiwan; 3 Department of Otolaryngology, National Taiwan University Hospital, Hsinchu, Hsinchu, Taiwan; 4 National Taiwan University Cancer Center, Taipei, Taiwan

## Abstract

Mucins are heavily glycosylated proteins that play critical roles in the pathogenesis of tumour malignancies. Pancreatic ductal adenocarcinoma (PDAC) is characterised by the aberrant expression of mucins. However, the role of mucin (MUC) 20 in PDAC remains unclear. PDAC is usually surrounded by a dense fibrotic stroma consisting of an extracellular matrix and pancreatic stellate cells (PSCs). The stroma creates a nutrient-deprived, hypoxic, and acidic microenvironment, and promotes the malignant behaviours of PDAC cells. In this study, immunohistochemical staining demonstrated that high MUC20 expression correlated with poor progression-free survival and high local recurrence rate of PDAC patients (*n* = 61). The expression of MUC20 was induced by serum deprivation, hypoxia, and acidic pH in PDAC cells. MUC20 knockdown with siRNA decreased cell viability, as well as migration and invasion induced by PSCs in HPAC and HPAF-II cells. In intraperitoneal, subcutaneous, and orthotopic injection models, MUC20 knockdown decreased tumour growth in immunodeficient mice. Phospho-RTK array and western blot analysis indicated that MUC20 knockdown decreased HGF-mediated phosphorylation of MET in PDAC cells. Moreover, HGF-induced malignant phenotypes could be suppressed by MUC20 knockdown. Co-immunoprecipitation revealed the physical association of MUC20 and MET. These findings suggest that MUC20 knockdown suppresses the malignant phenotypes of PDAC cells at least partially through the inhibition of the HGF/MET pathway and that MUC20 could act as a potential therapeutic target.

## Introduction

Pancreatic cancer is the ninth most common cancer in the western world, with a high mortality rate that ranks it as the fourth leading cause of cancer-related death [1]. Most cases of pancreatic cancer (>95%) are pancreatic ductal adenocarcinoma (PDAC) [2]. Since PDAC is usually resistant to chemotherapy and radiotherapy, the only potentially curative treatment for PDAC is surgical resection. However, <20% of patients are eligible for surgery, because most cases are at an advanced stage at the time of diagnosis [3]. Even though patients are eligible for surgery, 70–85% will experience disease recurrence after surgery. The consequence is an overall 5-year survival rate of PDAC patients that has remained <7% for 30 years [4, 5]. This disappointing survival rate highlights the urgency of understanding the molecular mechanisms of pancreatic cancer progression.

With the advance of knowledge and technique, cancer treatments have improved and better curative outcomes have been realised for many types of cancers, including melanoma, lung, and colorectal cancer [6]. Unfortunately, the same success has not been realised for PDAC. Several lines of evidence have provided insights into the influences of the microenvironment on the chemoresistance and radioresistance of cancer cells [7]. PDAC bulks are usually surrounded by thick fibrotic stroma, also called desmoplasia, which comprises up to 80% of the tumour mass [8]. The thick stroma plays a critical role in protecting PDAC cells from recent therapies and creates a nutrient-deprived, hypoxic, and acidic microenvironment. In addition, it is increasingly understood that the desmoplastic portion plays an active role in carcinogenesis, progression, metastasis, and immunosuppression [9]. The cellular portion of the desmoplastic stroma is considered to originate from pancreatic stellate cells (PSCs) normally located in the peri-acinar space. PSCs can be stimulated by alcohol, cytokines, and growth factors [10]. However, clearing desmoplastic stroma and PSCs leads to more aggressive cancers in animal models [11, 12]. Further knowledge of tumour-stromal interactions will help to develop novel therapeutic approaches and lead to new treatment strategies for PDAC patients, rather than the pure depletion approach.

Mucins are heavily glycosylated proteins. PDAC is characterized by the aberrant expression of both transmembrane and secretory mucins. The abnormal expressions of MUC4, MUC5AC, MUC5B, MUC13, MUC15, MUC16, and MUC17 are associated with disease progression in the pancreatic malignant precursor, pancreatic intraepithelial neoplasia (PanIN), and subsequent metastasis [13–18]. MUC1, MUC4, MUC5AC, and MUC16 have been associated with the progression, poor prognosis, and chemo-resistance of human pancreatic cancer. Moreover, mucins have been explored as candidates for cancer vaccines [19, 20] and therapeutic targets [21, 22]. MUC1-based therapies are now in clinical trial [23]. Although MUC20 has been reported to play important roles in endometrial and ovarian cancers [24, 25] and modulate the MET signalling cascade in IgA nephropathy [26, 27], the function and expression of MUC20 in PDAC remain unclear.

## Results

### MUC20 is overexpressed in PDAC and MUC20 high expression correlates with poor survival and high local recurrence rate

**Fig. 1:**
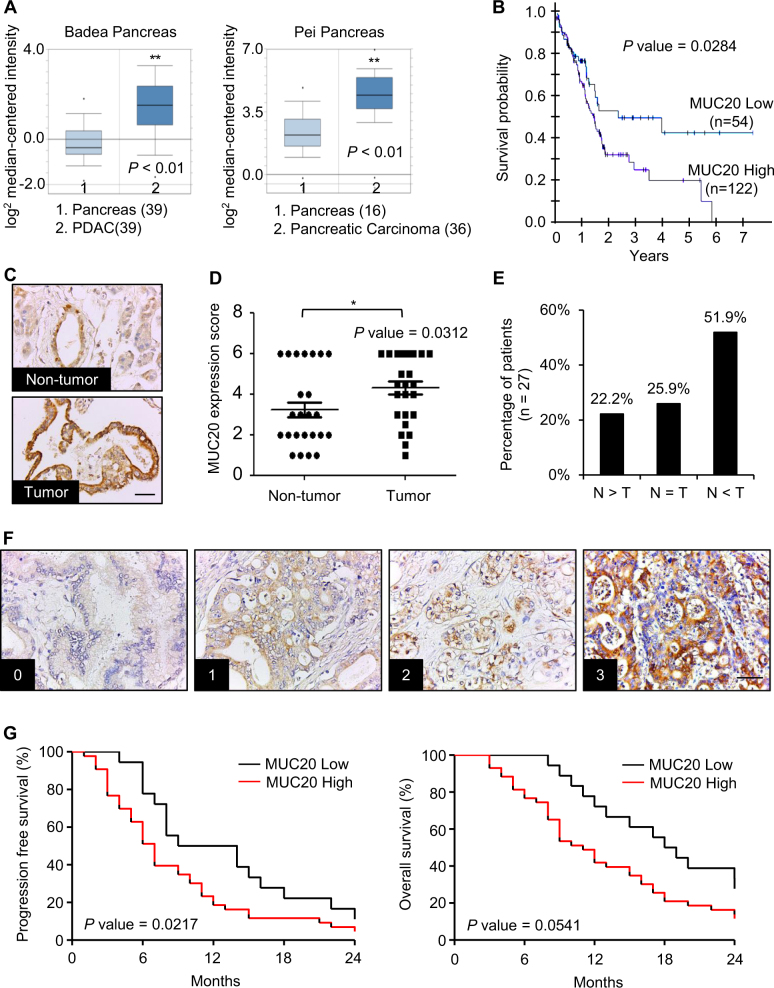
MUC20 is overexpressed in PDAC and MUC20 high expression correlates with poor survival. **a** *MUC20* mRNA levels in Badea Pancreas and Pei Pancreas from the Oncomine database. **b** Correlation between *MUC20* mRNA expression level and pancreatic cancer patient survival generated by the Cancer Genome Atlas (TCGA). *MUC20* low (FPKM ≤ 6) and high (FPKM > 6) expression group contained 54 and 122 patient samples, respectively. **c** Representative images showing MUC20 overexpression in pancreatic tumour tissues compared with the adjacent non-tumour tissue by immunohistochemistry (IHC) of tissue microarray (US Biomax, Inc). Scale bar indicates 50 μm. **d** Scatter plot graph represents the MUC20 expression score in non-tumour and tumour portions of the pancreas. MUC20 expression was scored by multiplication of intensity (0–3) and positive area (1–3). Data are presented as mean (*n* = 27) ± SEM. *P*-value was the result of the paired *t*-test. *P < 0.05. **e** Statistics of MUC20 expression in paired PDAC tissue microarray. Abbreviations are: N adjacent non-tumour, T tumour. **f** Representative images showing the intensity of MUC20 expression in PDAC tumours. Scale bar indicates 100 μm. **g** Kaplan–Meier plots of progression-free survival (left) and overall survival (right). Log-rank test, *P* = 0.0217 and 0.0541, respectively. MUC20 low and high expression were scored 0–5 (*n* = 18) and scored 6–9 (*n* = 43), respectively

Scrutiny of the Oncomine database determined that both the Pei Pancreas and Badea Pancreas feature higher *MUC20* mRNA expression in pancreatic carcinoma tissue compared with normal pancreas tissue (*P* < 0.01, Fig. 1a). In addition, TCGA RNA-seq data revealed that high *MUC20* mRNA expression correlated with poor survival (*P* *=* 0.0284, Fig. 1b). Immunohistochemistry (IHC) revealed the weak expression of MUC20 in pancreatic ductal cells in non-tumour regions. In contrast, MUC20 was expressed on the apical surface and cytoplasm of adenocarcinoma cells (Supplementary Fig. S1). The IHC of tissue microarray confirmed that MUC20 was overexpressed in pancreatic tumours compared with the adjacent non-tumour tissue (*P* *<* 0.05, Fig. 1c–e). Next, we analysed MUC20 expression using IHC in PDAC and correlated the expression with clinicopathologic characteristics and the prognosis of patients. The IHC score of MUC20 was the product of the staining intensity (0–3) (Fig. 1f) multiplied by positive area (1–3). Chi-square statistics showed that high MUC20 expression correlated with high local recurrence rate (Table 1). Moreover, Kaplan–Meier survival curves indicated that MUC20 high expression (scores 6–9) correlated with poor progression-free survival (*P* = 0.0217, Fig. 1g). Taken together, the data indicate that MUC20 is overexpressed in PDAC at mRNA and protein levels compared with normal pancreas, and that the higher expression of MUC20 is associated with poorer prognosis.

**Table 1:** Association between MUC20 expression with clinicopathologic characteristics of PDAC

**Characteristics**	**MUC20 expression (n** ^**a**^ **)**		***P*** **value** ^**b**^
	**Low**	**High**	
Age (years)			0.548
≤60	7	9	
>60	15	30	
Gender			0.173
Male	17	23	
Female	5	16	
Differentiated grades			0.342
Better	19	29	
Poor	3	10	
Lymph node metastasis			0.416
No	11	14	
Yes	11	25	
Distant metastasis			0.588
No	9	13	
Yes	13	26	
AJCC stages			0.251
Early stage^c^	10	12	
Late stage	12	27	
Local recurrence			0.026*
No	9	6	
Yes	13	33	
Adjuvant therapy			0.426
No	14	20	
Yes	8	19	

^a^number of case

^b^Chi-square test

^c^early stage indicates AJCC stage IB and IIA; late stage indicates ATCC stage IIB, III, and IV

^*^P < 0.05

### MUC20 knockdown inhibits 10% foetal bovine serum (FBS)-induced pancreatic cancer cell viability, but not induced migration and invasion

**Fig. 2:**
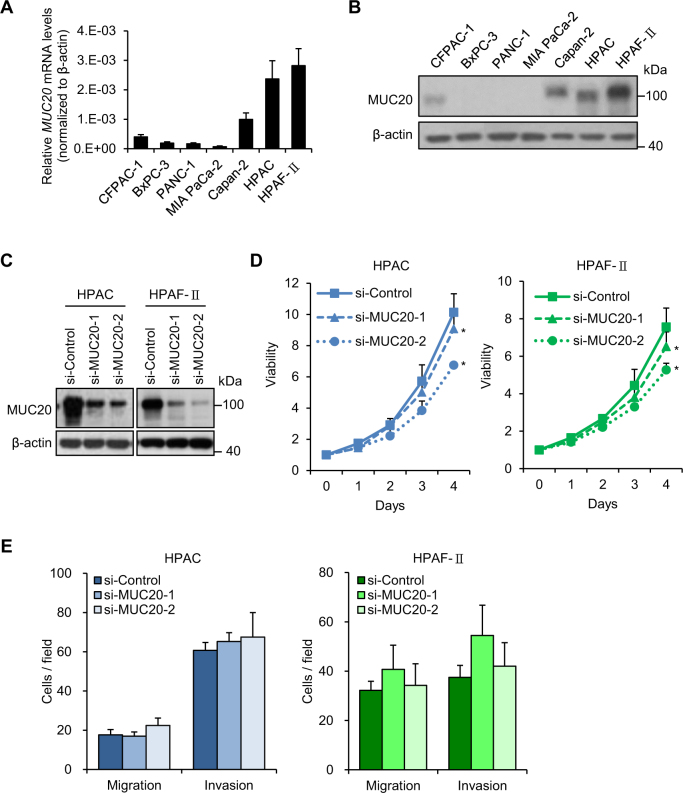
Effects of MUC20 knockdown on PDAC cells in vitro. **a** The mRNA levels of *MUC20* analysed by real-time RT-PCR in PDAC cell lines, as indicated. **b** The protein levels of MUC20 analysed by Western blotting in PDAC cell lines. **c** Western blots showing MUC20 knockdown with two independent siRNAs (si-MUC20-1 and si-MUC20-2) in HPAC and HPAF-II cells. **d** MUC20 knockdown inhibited viability in HPAC and HPAF-II cells analysed by MTT assays. *P < 0.05 **e** MUC20 knockdown did not significantly affect 10% FBS-triggered migration and invasion in HPAC and HPAF-II cells analysed by Transwell migration and Matrigel invasion assay, respectively. Data are presented as mean (*n* = 3) ± SD

Real-time RT-PCR (Fig. 2a) and western blot analysis (Fig. 2b) revealed variations in the expression levels of MUC20 in the seven PDAC cell lines. MUC20 was knocked down in HPAC and HPAF-II cells, which express higher MUC20, using two independent small interfering (si)RNAs (Fig. 2c). To assess the effects of MUC20 on PDAC cells, 10% FBS-induced viability, migration, and invasion of PDAC cells were analysed by 3-(4,5-dimethylthiazol-2-yl)-2,5-diphenyltetrazolium bromide (MTT), Transwell migration, and Matrigel invasion assays, respectively. MUC20 knockdown significantly suppressed viability in both HPAC and HPAF-II cells (Fig. 2d). However, no significant changes in migration and invasion were observed (Fig. 2e). These results suggest that MUC20 knockdown inhibits 10% FBS-induced viability, but not migration and invasion, in PDAC cells.

### MUC20 knockdown suppresses PDAC tumour growth in immunodeficient mice

**Fig. 3:**
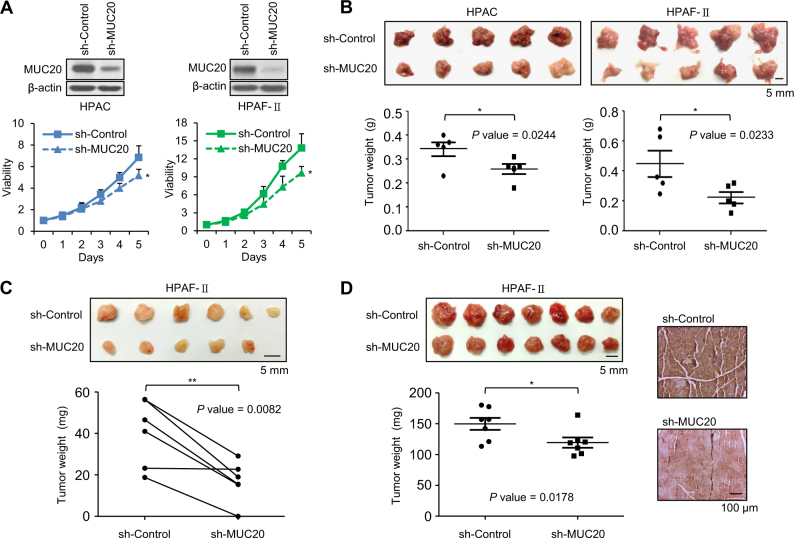
MUC20 knockdown suppresses PDAC tumour growth in immunodeficient mice. **a** Western blots showing stable MUC20 knockdown in HPAC and HPAF-II cells. MUC20 knockdown inhibited viability in HPAC and HPAF-II cells analysed by MTT assays. Cells were stably transfected with MUC20 shRNA (sh-MUC20) or non-targeting control shRNA (sh-Control). Data are presented as mean (*n* = 3) ± SD. **b** MUC20 knockdown decreased tumour sizes (upper) and weights (lower) compared with the control group (*n* = 5 for each group) after four weeks of intraperitoneal injection with HPAC or HPAF-II cells in nude mice. Data are presented as mean ± SEM. **c** MUC20 knockdown decreased tumour sizes (upper) and weights (lower) compared with the control group (*n* = 6 for each group) after four weeks of subcutaneous injection with HPAF-II cells in NOD/SCID mice. **d** The left panel shows MUC20 knockdown decreased tumour sizes (upper) and weights (lower) compared with the control group (*n* = 7 for each group) after four weeks of orthotopic injection with HPAF-II cells in NOD/SCID mice. The right panel shows representative images of IHC for MUC20 in control and MUC20 knockdown tumours. Data are presented as mean ± SEM. *P < 0.05; **P < 0.01

To investigate the effect of MUC20 on PDAC tumour growth, HPAC and HPAF-II cells were xenografted in immunodeficient mice. The stable knockdown of MUC20 with short hairpin (sh)RNA in HPAC and HPAF-II cells was confirmed by western blotting, and viability was analysed using the MTT assay (Fig. 3a). After four weeks of intraperitoneal injection, tumours were present in the abdominal cavity. MUC20 knockdown tumours were smaller and weighed less than control tumours for both HPAC (*P* = 0.0244) and HPAF-II cells (*P* = 0.0233) (Fig. 3b). In the subcutaneous injection model, MUC20 knockdown decreased the sizes and weights of HPAF-II tumour cells compared with the control group in NOD/SCID mice (*P* = 0.0082, Fig. 3c). Moreover, MUC20 knockdown decreased tumour formation after four weeks of orthotopic injection with HPAF-II cells in NOD/SCID mice. Immunohistochemistry confirmed that the higher MUC20 expression was observed in excised control tumour than that in MUC20 knockdown tumour (*P* = 0.00178, Fig. 3d). These findings suggest that MUC20 knockdown inhibits PDAC tumour growth in immunodeficient mice.

### MUC20 is up-regulated in the serum-deprived, hypoxic, and acidic microenvironment

**Fig. 4:**
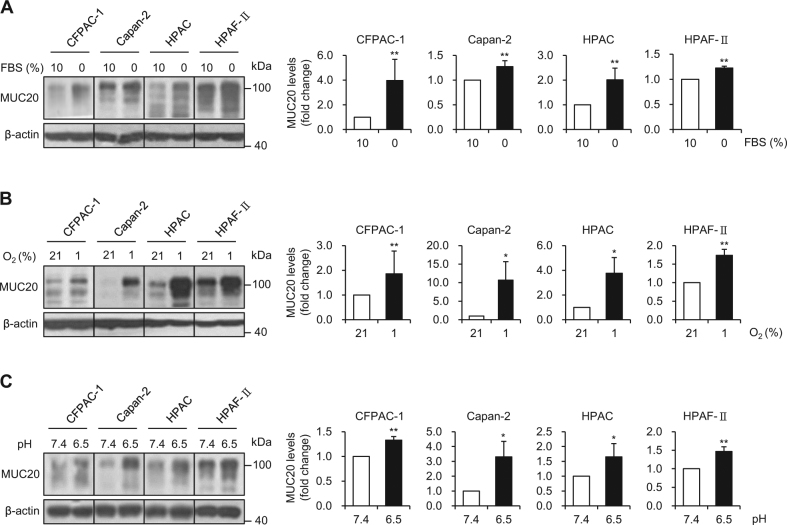
MUC20 is up-regulated in serum-deprived, hypoxic, and acidic microenvironment. **a** MUC20 was induced by serum deprivation (0% FBS). **b** MUC20 was induced by hypoxia (1% oxygen). **c** MUC20 was induced by acidic condition (pH 6.5). PDAC cells were treated with these different microenvironmental factors for 24 h. The expression of MUC20 was analysed by western blotting. β-actin was used as an internal control. Statistical results for MUC20 signals are shown. Data are presented as mean (*n* = 3) ± SD. *P < 0.05; **P < 0.01

Since the microenvironment plays a critical role in PDAC progression, we examined whether MUC20 expression could be modulated by microenvironmental factors including serum-deprivation, hypoxia, and acidic pH. PDAC cells were treated with these factors for 24 h and then MUC20 expression was analysed by western blotting. MUC20 was upregulated by serum-deprivation (Fig. 4a), hypoxia (Fig. 4b), and acidic pH (Fig. 4c) in CFPAC-1, Capan-2, HPAC, and HPAF-II cells. A low concentration (1%) of FBS was sufficient to induce MUC20 expression (Supplementary Fig. S2). Interestingly, the mRNA level of *MUC20* was upregulated by serum deprivation in HPAC and HPAF-II cells (Supplementary Fig. S3A). Serum deprivation increased the activity of phospho-c-Jun N-terminal kinase (p-JNK), but not p-p38 (Supplementary Fig. S3B). Inhibition of p-JNK activity using SP600125 could suppress MUC20 expression induced by serum deprivation (Supplementary Fig. S3C), suggesting that the p-JNK signalling pathway is involved in the MUC20 induction by serum deprivation. These results suggest that MUC20 expression can be induced by tumour microenvironmental factors in PDAC cells, which include CFPAC-1, Capan-2, HPAC, and HPAF-II cell lines.

### MUC20 knockdown inhibits migration and invasion of PDAC cells co-cultured with PSCs

**Fig. 5:**
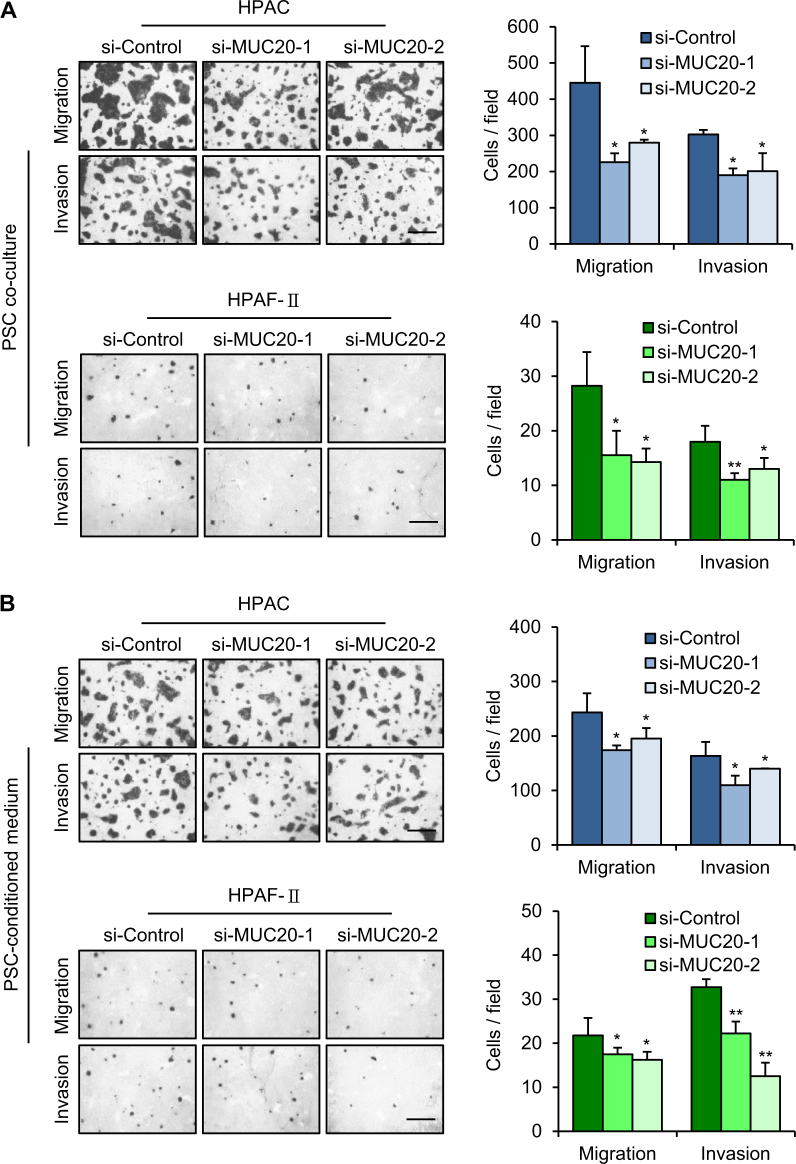
MUC20 knockdown inhibits migration and invasion of PDAC cells co-cultured with PSCs. **a** MUC20 knockdown inhibited migration and invasion induced by pancreatic stellate cells (PSCs) in HPAC (upper) and HPAF-II (lower) cells. **b** MUC20 knockdown inhibited migration and invasion induced by PSC-conditioned medium. Cell migration and invasion were analysed by Transwell migration and Matrigel invasion assay, respectively. The left panel shows representative images of migrated and invaded cells. The right panel shows statistics of cell migration and invasion. Scale bars indicate 1 mm. Data are presented as mean (*n* = 3) ± SD. *P < 0.05; **P < 0.01

PDAC is characterized by its dense surrounding connective tissue resulting in the stromal barrier. This dense stromal barrier may be one of the possible leading causes of the nutrient-deprived, hypoxic, and acidic microenvironment in PDAC. Given that PSCs are the main cellular source of the dense stroma of PDAC, we were interested in the communication between PSCs and PDAC cells. We co-cultured PDAC cells with PSCs in Dulbecco’s modified Eagle’s medium (DMEM)-F12 containing 1% FBS and then performed Transwell migration and Matrigel invasion assays. MUC20 knockdown significantly decreased migration and invasion induced by PSC co-culture in both HPAC and HPAF-II cells (Fig. 5a). Next, we cultured PSCs in DMEM-F12 containing 1% FBS for 24 h and then collected the conditioned medium to trigger PDAC cell migration and invasion. MUC20 knockdown significantly suppressed migration and invasion induced by the PSC-conditioned medium in both HPAC and HPAF-II cells (Fig. 5b). However, MUC20 knockdown did not significantly affect 1% FBS-triggered migration and invasion without PSCs (Supplementary Fig. S4). These results suggest that MUC20 knockdown inhibits migration and invasion of PDAC cells during PSC co-culture or in PSC-conditioned medium.

### MUC20 enhances hepatocyte growth factor (HGF)/MET signalling in PDAC cells

**Fig. 6:**
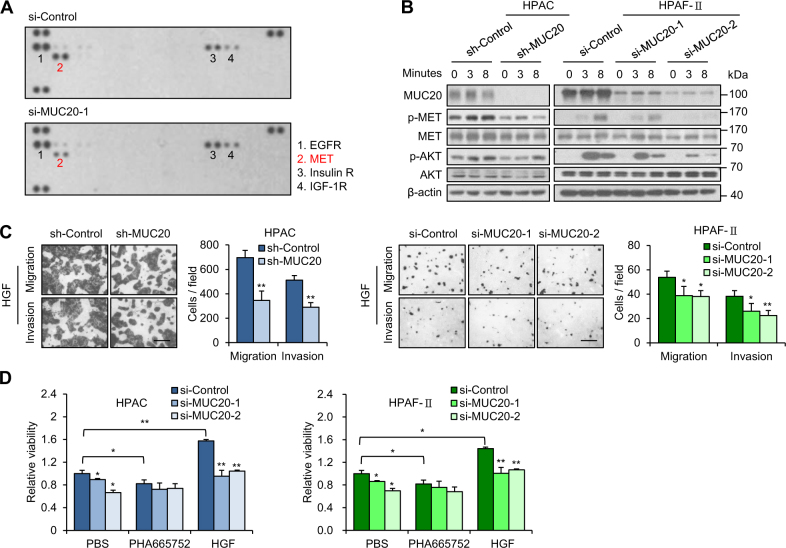
MUC20 knockdown inhibits HGF/MET signalling in PDAC cells. **a** MUC20 knockdown decreased phospho-MET levels. MUC20 was knocked down with siRNA (si-MUC20-1) in HPAF-II cells cultured with PSC-conditioned medium. Changes in 49 phospho-RTKs were analysed by western blotting of human phospho-RTK array (R&D Systems). **b** MUC20 knockdown inhibited phosphorylation of MET and AKT in HPAC and HPAF-II cells treated with 25 ng/ml HGF. **c** MUC20 knockdown inhibited HGF-triggered migration and invasion in HPAC and HPAF-II cells analysed by Transwell migration assay and Matrigel invasion assay, respectively. Scale bars indicate 1 mm. **d** MUC20 knockdown significantly reversed HGF-enhanced viability in HPAC and HPAF-II cells. Cells cultured in 10% FBS/DMEM-F12 were treated with PBS, MET inhibitor PHA665752 (2.5 µM) or HGF (25 ng/ml), as indicated. Viability was analysed by the MTT assay. Data are presented as mean (*n* = 3) ± SD. *P < 0.05; **P < 0.01

Since mucins have been reported to regulate cellular phenotypes through various receptor tyrosine kinases (RTKs), we first analysed the effects of MUC20 on RTK signalling pathways. Results from a human phospho-RTK array showed that MUC20 knockdown decreased phospho-MET levels in HPAF-II cells induced by the PSC-conditioned medium (Fig. 6a). Interestingly, increased mRNA levels of *HGF*, a ligand of MET, were also observed in PSCs treated with 1% FBS for 24 h (Supplementary Fig. S5). Next, we investigated AKT, extracellular signal-regulated kinase (ERK), and nuclear factor-kappa B (NF-kB) activities, which have been reported to be important in downstream signalling pathways of MET [28, 29]. Western blotting results showed that MUC20 knockdown inhibited HGF-triggered phosphorylation of MET and AKT in HPAC and HPAF-II cells (Fig. 6b, Supplementary Fig. S6A). However, ERK and NF-kB activities were not affected by MUC20 knockdown (Supplementary Fig. S6B). By contrast, overexpression of MUC20 increased HGF-triggered phosphorylation of MET and AKT in HPAF-II cells (Supplementary Fig. S7A). In addition, we investigated the effect of MUC20 on PDAC cellular phenotypes triggered by HGF in vitro. The Transwell migration assay and Matrigel invasion assay revealed that MUC20 knockdown decreased HGF-induced cell migration and invasion (Fig. 6c), whereas overexpression of MUC20 enhanced HGF-induced cell migration (Supplementary Fig. S7B). To investigate the role of the MET signalling pathway in MUC20-mediated cell viability, we treated PDAC cells with the MET inhibitor PHA665757 or HGF. MTT assays showed that MUC20-mediated cell viability was significantly inhibited by PHA665757, but was enhanced by HGF (Fig. 6d). Furthermore, we analysed the role of AKT in phenotypic changes mediated by MUC20. AKT overexpression increased the HGF-triggered viability, migration, and invasion in MUC20 knockdown cells in the MTT, Transwell migration and Matrigel invasion assays, respectively (Supplementary Fig. S8). Taken together, these results suggest that MUC20 enhances the HGF/MET signalling pathway in PDAC cells.

### Physical interactions of MUC20 and MET occur in PDAC cells

**Fig. 7:**
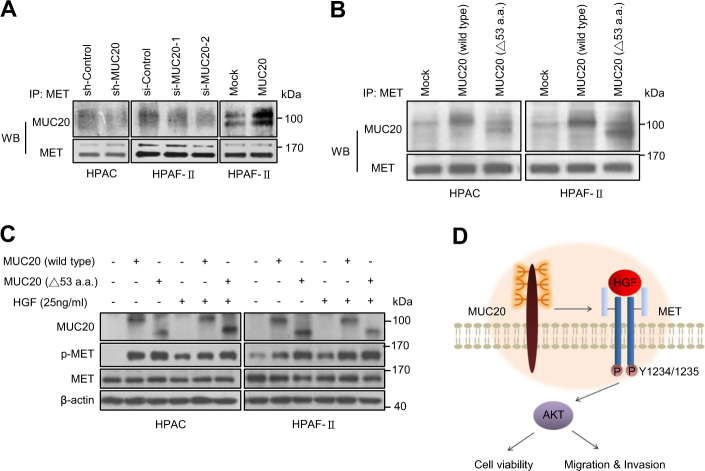
Physical interactions of MUC20 and MET occur in PDAC cells. **a** Physical interactions of MUC20 with MET were revealed by co-immunoprecipitation assay (Co-IP). Whole cell lysates were immunoprecipitated (IP) with anti-MET antibody and then western blotted (WB) with anti-MUC20 or anti-MET antibody. **b** Truncated MUC20 still interacted with MET. Truncated MUC20 with a C-terminal deletion of 53 amino acids, MUC20 (△53 a.a.), was constructed. Co-IP assays showed that both wild-type and truncated MUC20 interacted with MET. **c** MUC20 overexpression enhanced phosphorylation of MET in HPAC and HPAF-II cells treated with 25 ng/ml HGF. The truncated MUC20 further increased p-MET compared with the wild-type MUC20. **d** Schematic diagram depicting how MUC20 mediates its biological effects through MET in PDAC cells

To analyse the interaction of MUC20 with MET, we performed a co-immunoprecipitation assay. MUC20 physically interacted with MET, and MUC20 knockdown decreased the association of MET with MUC20 in HPAC and HPAF-II cells (Fig. 7a). By contrast, overexpression of MUC20 increased their association in HPAF-II cells. It has been reported that the C terminal 53-amino acid region of MUC20 could be the binding domain of MET [26]. To examine whether MUC20 interacted with MET through this domain in pancreatic cancer cells, we constructed the same truncated MUC20. Interestingly, the results of the co-immunoprecipitation assay indicated that both wild-type and truncated MUC20 interacted with MET in HPAC and HPAF-II cells (Fig. 7b). In addition, western blotting results showed that the truncated MUC20 further enhanced MET phosphorylation compared with the wild-type MUC20 (Fig. 7c). These results suggest that MUC20 physically interacts with MET in PDAC cells and the binding is independent of its C-terminal 53-amino acid domain.

## Discussion

The most commonly used tumour marker, carbohydrate antigen 19-9 (CA 19-9), is not very accurate in PDAC detection [30, 31]. In addition, <20% of patients survive longer than 5 years after receiving surgical resection, which is the only curative treatment in PDAC [3]. Given these terrible circumstances in PDAC, a suitable biomarker and treatment are urgently needed. MUC1 is the most investigated mucin in PDAC, and many approaches, including vaccines, drugs, and antibodies, are being developed to target MUC1. However, agents that target MUC1 in these approaches fail to reach the surface of the cancer cells because of the abundant expression of MUC1 in circulation and normal tissues [32]. Compared to MUC1, public databases show that MUC20 expression in human tissues is lower and is more tissue-specific. In addition, this study proves that MUC20 expression is upregulated in PDAC tissues compared with that in non-tumour pancreas tissues, and that the high expression of MUC20 correlates with poor survival. These results implicate MUC20 as a potential marker to distinguish benign from malignant pancreatic tissue. Furthermore, MUC20 knockdown inhibits tumour cell growth both in vitro and in vivo. This study is the first to suggest that MUC20 plays a critical role in PDAC pathogenesis and could be a potential target for biomarker and drug development.

PDAC is usually surrounded by a dense fibrotic stroma consisting of extracellular matrix proteins and PSCs. The stroma is thought to influence carcinogenesis, progression, and metastasis [7, 8]. In this study, MUC20 knockdown decreased migration and invasion of PDAC cells induced by PSCs and PSC-conditioned medium. These findings suggest that MUC20 enhances PDAC malignant behaviours by modulating factors secreted by PSCs. HGF/MET signalling has been repeatedly reported to be a critical pathway for communication between stroma and cancer cells. PDAC-derived PSCs enhance proliferation, migration, and invasion of PDAC cells by secreting HGF [33–36]. In this study, MUC20 knockdown inhibited the phospho-MET activity triggered by PSC-conditioned medium and recombinant HGF in PDAC cells. Additionally, MUC20-increased cell viability was inhibited by a MET inhibitor, but was increased by HGF. These data support the view that MUC20 can enhance the malignant behaviours at least partly through the HGF/MET signalling pathway in PDAC cells.

It is worth noting that the HGF secretion of PSCs can be induced by microenvironmental factors including hypoxia [37, 38] and serum deprivation (Supplementary Fig. S5). Interestingly, we observed that MUC20 expression was also upregulated by PDAC microenvironments, such as nutrient-deprivation, hypoxia, and acidic pH. These findings suggest that tumour microenvironmental factors could induce both HGF and MUC20 expression and, in turn, enhance the MUC20/HGF/MET signalling pathway to promote PDAC progression. However, since MUC20 expression can be upregulated by serum deprivation, it is important to know the status of MUC20 levels in control and MUC20 knockdown cells under serum deprivation. We showed that the MUC20 expression was elevated in both the control and MUC20 knockdown cells under the condition of serum deprivation (Supplementary Fig. S9). The difference in the level of MUC20 expression between these two cells still existed. Therefore, the effects of MUC20 on phenotypes could be assessed although MUC20 expression was induced under serum deprivation conditions.

This study indicates that MUC20 enhances the HGF-induced phosphorylation of MET and AKT in PDAC cells. By contrast, Toshio Higuchi et al. found that MUC20 suppresses HGF-induced Grb2/p-ERK, but not p-MET/p-AKT activity, in primary normal renal cells [26]. They further demonstrated that the major binding region of MUC20 with MET is primarily located at the C-terminal 53-amino acid domain. Unexpectedly, we found that the truncated MUC20 still interacted with MET in PDAC cells without a loss of binding activity. This discrepancy could have resulted from the differences between normal and cancer cells or different cell types. To well understand the MET signalling pathways modulated by MUC20, identification of the binding site between MUC20 and MET is required.

In conclusion, MUC20 is frequently up-regulated in PDAC tumours compared with non-tumour pancreas tissue, and MUC20 high expression correlates with poor prognosis of patients. MUC20 knockdown decreases PDAC tumour growth in immunodeficient mouse models. Furthermore, PSC-mediated malignant phenotypes are inhibited by MUC20 knockdown in PDAC cells through the HGF/MET signalling pathway. Our results suggest that MUC20 physically interacts with MET and enhances HGF-mediated phosphorylation of MET and AKT, thereby promoting malignant phenotypes of PDAC cells (Fig. 7d). These findings demonstrate that MUC20 is a novel regulator of PDAC malignant behaviours triggered by HGF or PSCs and implicate MUC20 as a potential diagnostic marker and therapeutic target.

## Materials and methods

### Immunohistochemistry

Pancreatic adenocarcinoma tissue microarray with matched cancer adjacent tissues (Biomax PA811) was purchased from US Biomax, Inc. (Rockville, MD, USA) for immunohistochemical staining. Tissue slides of 61 PDAC patients with different histological grades and stages were obtained from National Taiwan University Hospital with IRB approval (201411085RINB). Polyclonal anti-MUC20 antibody made by our laboratory [24] was used to recognize MUC20 protein and the signal was detected by UltraVision Quanto Detection System (Thermo Scientific, Cheshire, UK). MUC20 expression was scored by multiplication of intensity (0–3) and positive area (1–3). Intensities were scored as 0 (negative), 1 (faint), 2 (moderate), and 3 (strong). Positive areas were scored as 1 (<33%), 2 (33–66%), and 3 (>66%). MUC20 low and high expression were scored 0–5 and 6–9, respectively.

### Cell lines and cell culture

Human pancreatic cancer cell lines, CFPAC-1, MIA PaCa-2, PANC-1, Capan-2, HPAC, and HPAF-II, and pancreatic stellate cell line, PSC, were kindly provided by Dr. WH Lee (Genomics Research Center, Academia Sinica, Taiwan). All cell lines had been further confirmed by DNA typing. All cell lines were cultured in medium containing 10% FBS (Gibco, Gaithersburg, MD, USA), 100 IU/mL penicillin, and 100 μg/mL streptomycin (Gibco) in a humidified tissue culture incubator at 37 °C and 5% CO_2_ atmosphere. Dulbecco’s modified Eagle’s medium (DMEM; Invitrogen, Grand Island, NY, USA) was used for MIA PaCa-2 and PANC-1 cells. DMEM-F12 GlutaMAX^TM^ (Invitrogen) was used for CFPAC-1, Capan-2, HPAC, HPAF-II, and PSC cells.

### cDNA synthesis and real-time RT-PCR

Total RNA was isolated using TRIzol reagent (Invitrogen) according to the manufacturer’s protocol. For cDNA synthesis, 2 µg of total RNA was used in a 20 µl reverse transcription reaction using the High-Capacity cDNA Reverse Transcription Kits (Applied Biosystems, Foster City, CA, USA). The cDNA was subjected to real-time PCR using QuantStudio 3 Real-Time PCR System (Thermo Fisher Scientific, Waltham, MA, USA). The real-time PCR reactions were performed in 20-µl volume containing 1 µl cDNA, 10 µl SensiFAST SYBR Lo-ROX Mix (Bioline, London, UK) and primer pairs. The following primer pairs were used: *β-actin* sense, 5′-CGTGCGTGACATTAAGGAGA-3′ and anti-sense, 5′-GAAGGAAGGCTGGAAGAGTG-3′; *MUC20* sense, 5′-AACTCCACGCCCACGCGCCT-3′ and anti-sense, 5′-GGAAGCACACAGATGGGTG-3′; *HGF* sense, 5′-ATGATGTCCACGGAAGAGGAGA-3′ and anti-sense, 5′-CACTCGTAATAGGCCATCATAGTTGA -3′.

### Transfection and plasmid construction

For transient MUC20 knockdown, two independent siRNAs and non-targeting siRNA (Dharmacon, ThermoFisher Scientific, MA, USA) were used to transfect PDAC cells by Lipofectamine RNAiMAX (Invitrogen) with a final concentration of 10 nM for 3 days. For stable MUC20 knockdown and its control cells, sh-MUC20/pLKO.1 plasmid and pLKO.1 vector (RNAi Core, Academia Sinica, Taiwan) were used in lentivirus-based infection system, respectively, and selected with 2 μg/ml puromycin (Sigma. St. Louis, MO, USA). MUC20 overexpression and its mock control cells were established by transfection of MUC20/pcDNA3.1 A plasmid or pcDNA3.1 A vector, respectively, using Lipofectamine 3000 (Invitrogen) according to the manufacturer’s protocol. Human wild-type *MUC20* (NCBI Accession No. NM_001282506.1) and truncated *MUC20* were cloned using PCR kit (Invitrogen). The sense primer was 5′-AAGCTTATGGGCTGTCTCTGGGGTCT-3′. Antisense primer for wild-type *MUC20* was 5′-GGATCCTTAGCCTCTCCTGACACGCA-3′. Antisense primer for truncated *MUC20* was 5′-GGATCCTTATGCACTCACGTCTGTGGTC-3′. The PCR products were cloned into pcDNA3.1/myc-His (Invitrogen) to generate the MUC20/pcDNA3.1A plasmid. The MUC20 was confirmed by DNA sequencing. AKT/PCIS2 plasmid and its control vector, PCIS2, were gifts from Dr. Michael J. Quon (University of Maryland School of Medicine, Division of Endocrinology, USA).

### Antibodies and reagents

MUC20 antibody was prepared as described in our previous study [24]. Antibody against β-actin (A5441) was obtained from Sigma. Antibodies against MET (GTX100637), AKT (GTX121937), NFκB (GTX102090), and p-NFκB (GTX50098) were purchased from GeneTex Inc. (Irvine, CA, USA). Antibodies for immunoprecipitation of MET (#8198) and for MET pY1234/5 (#3077), p-AKT (#4060), ERK (#9102), and p-ERK (#9101) were purchased from Cell Signaling Technology, Inc. (Danvers, MA, USA). Recombinant HGF was purchased from Sigma. PHA665752, MET inhibitor, was purchased from Tocris Bioscience (Bristol, UK). SP600125, JNK inhibitor, was purchased from Selleckchem (Houston, TX, USA).

### MTT assay

Pancreatic cancer cells (1.5 × 10^3^) in 100 μl complete DMEM-F12 were seeded in 96-well plates for 16 h. Ten microliters of 5 mg/ml 3-(4,5-dimethyl-2-thiazolyl)-2,5-diphenyl-2H-tetrazolium bromide solution (MTT; Sigma) was added to each well for the indicated times and incubated at 37 ^o^C for 3 h, and the MTT formazan crystals were dissolved with 100 μl 10% SDS containing 0.01 N HCl. The resultant optical density was measured spectrophotometrically at dual wavelengths, 550 and 630 nm.

### Transwell migration and Matrigel invasion assays

Cell migration and invasion assays were evaluated with empty Transwell (Corning, NY, USA) or Matrigrl-coated (BD Biosciences, San Jose, CA, USA) Transwell chamber, respectively. Each Transwell chamber contains an 8-μm pore size membrane. Pancreatic cancer cells (5 × 10^4^) in 0.25 ml serum-free DMEM-F12 were seeded into the Transwell or Matrigel-coated Transwell chamber and then the chambers were put into 24-well plates loaded with 0.5 ml of 10% FBS, 1% FBS, 25 ng/ml HGF, PSCs or PSC-conditioned medium, respectively. After 48 h of incubation, cells were fixed and stained with 0.5% (w/v) crystal violet (Sigma) containing 20% (v/v) methanol. The number of migrated cells from 5 random fields was counted under the microscope.

### In vivo xenograft tumour growth model

For tumour growth analysis, stable transfectants were xenografted in 8 w/o female immunodeficient mice (National Laboratory Animal Center, Taiwan). 5 × 10^6^ of cells in 0.5 ml serum-free DMEM-F12 were intraperitoneally injected into each nude mouse. 5 × 10^6^ of cells in 100 µl serum-free DMEM-F12 containing 50% Matrigel (BD Biosciences) were subcutaneously injected into each NOD/SCID mouse. 10^6^ of cells in 50 µl serum-free DMEM-F12 were orthotopically injected into each NOD/SCID mouse. Animals were sacrificed after 30 days of tumour cell injection. All animal interventions were reviewed and approved by the Institutional Animal Care and Use Committee IACUC of College of Medicine, National Taiwan University.

### Phospho-receptor tyrosine kinase array assay

Human phospho-receptor tyrosine kinase (p-RTK) array kit including 49 RTKs was purchased from R&D systems (Minneapolis, MN, USA). HPAF-II cells were serum starved for 24 h and then stimulated with PSC-conditioned medium for 8 min. Cells were lysed and 500 μg of protein were subjected to western blotting according to the manufacturer’s protocol.

### Immunoprecipitation

For immunoprecipitation, 1000 μg of whole cell lysates were prepared and incubated with specific antibody for 16 h at 4 °C. After that, the reaction products were applied to Protein G agarose beads (GE Healthcare, Munich, Germany) at 4 °C for 3 h. Precipitated proteins were then analysed by western blotting.

### Statistical analysis

Statistics were performed using Prism 5 and SPSS 22.0 statistical software. Survival curves were plotted by Kaplan–Meier. The correlations between MUC20 expression and clinicopathologic characteristics were tested using Chi-square test. Student *t*-test was used to compare differences between two experimental groups. Data are presented as means (*n* = 3) ± SD and *P* < 0.05 was considered statistically significant.

## Electronic supplementary material

Supplementary figure 1

Supplementary figure 2

Supplementary figure 3

Supplementary figure 4

Supplementary figure 5

Supplementary figure 6

Supplementary figure 7

Supplementary figure 8

Supplementary figure 9

Supplementary Figure Legends
