# Supplementary figures and images for: Silencing of MUC20 suppresses the malignant character of pancreatic ductal adenocarcinoma cells through inhibition of the HGF/MET pathway
Source: Oncogene. 2018 Jul 11;37(46):6041–53. doi: 10.1038/s41388-018-0403-0 (PMC6237765; doi:10.1038/s41388-018-0403-0)

Supplementary figure S1

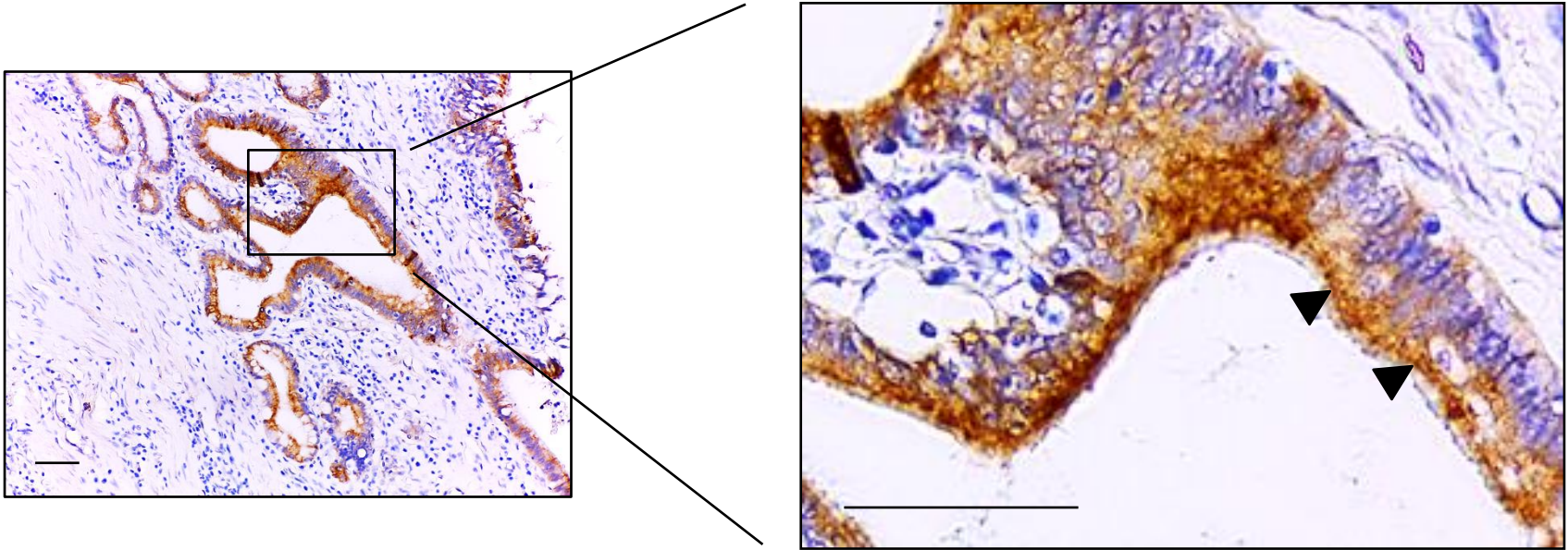

Supplement: Supplementary file 1 — Supplementary figure 1 [file 41388_2018_403_MOESM1_ESM.pdf]

Supplementary figure S2

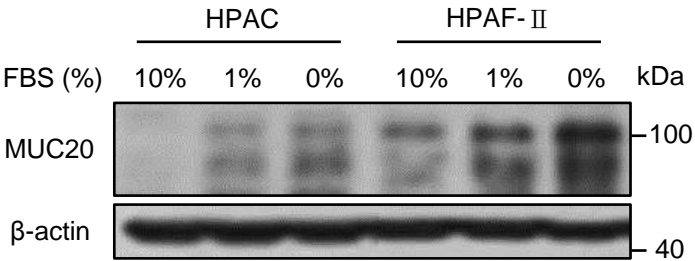

Supplement: Supplementary file 2 — Supplementary figure 2 [file 41388_2018_403_MOESM2_ESM.pdf]

Supplementary figure S3

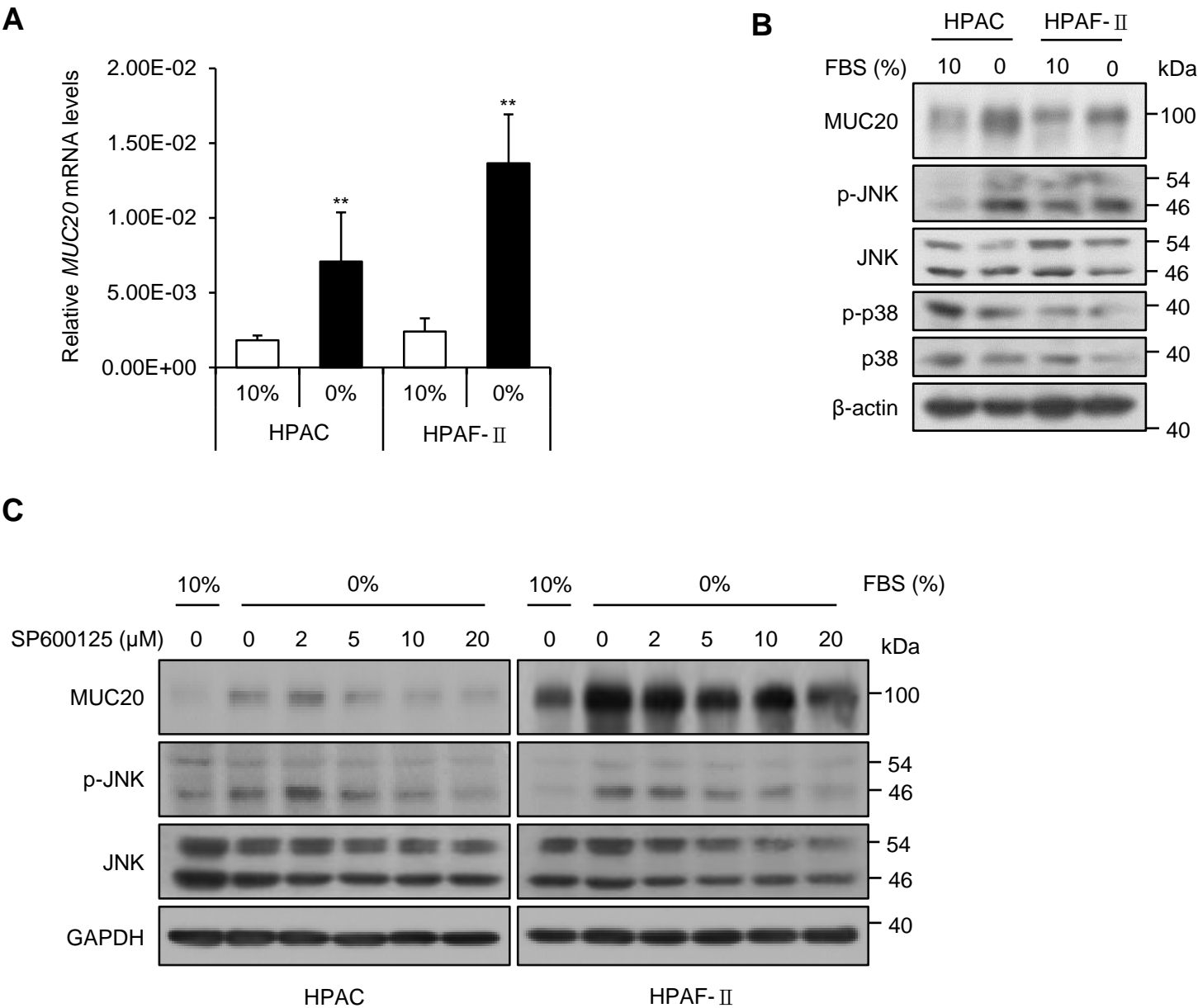

Supplement: Supplementary file 3 — Supplementary figure 3 [file 41388_2018_403_MOESM3_ESM.pdf]

Supplementary figure S4

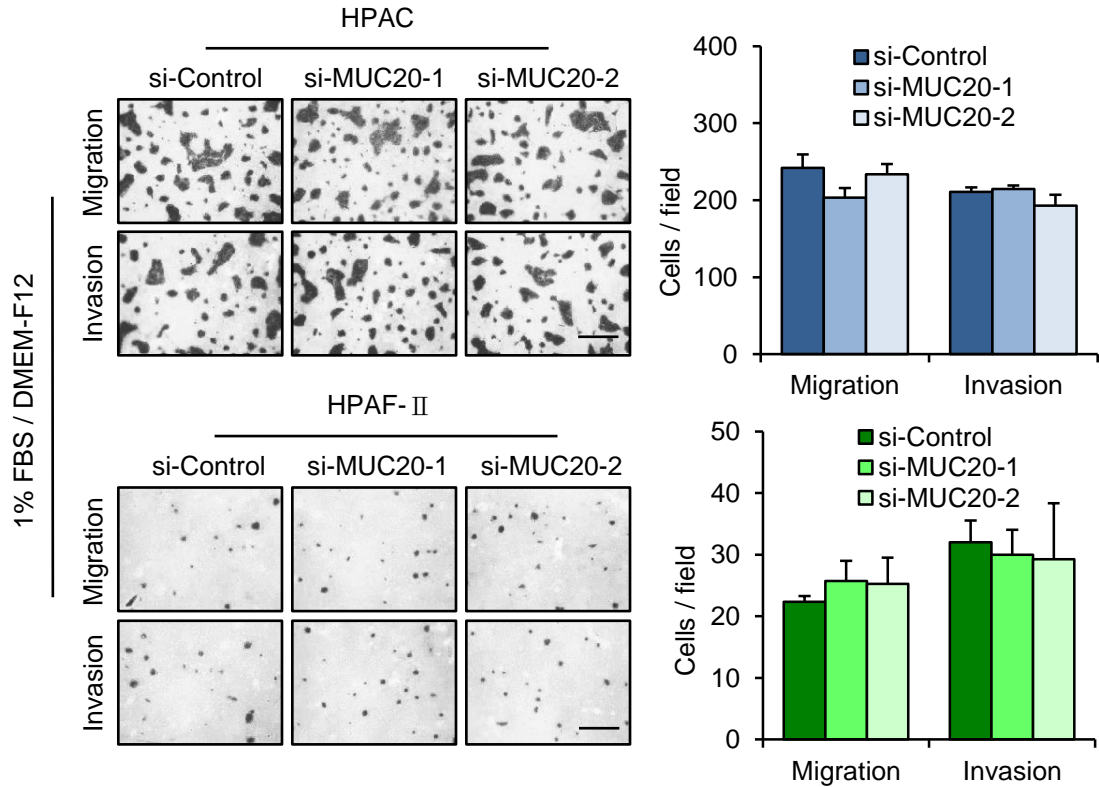

Supplement: Supplementary file 4 — Supplementary figure 4 [file 41388_2018_403_MOESM4_ESM.pdf]

Supplementary figure S5

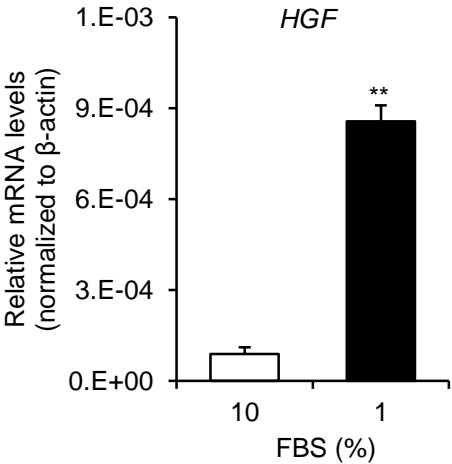

Supplement: Supplementary file 5 — Supplementary figure 5 [file 41388_2018_403_MOESM5_ESM.pdf]

Supplementary figure S6

**A**

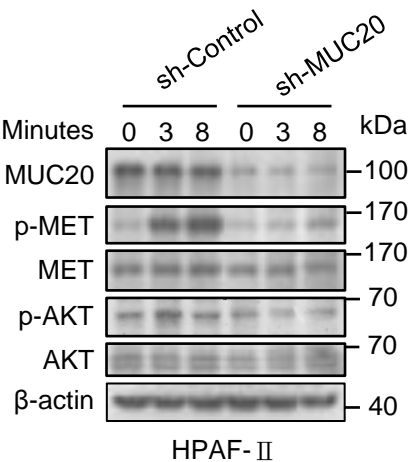

**B**

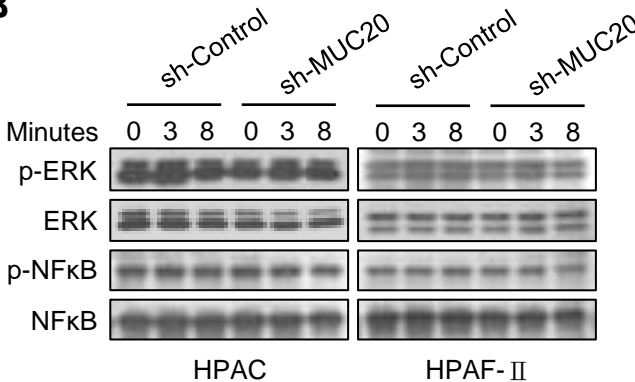

Supplement: Supplementary file 6 — Supplementary figure 6 [file 41388_2018_403_MOESM6_ESM.pdf]

Supplementary figure S7

**A**

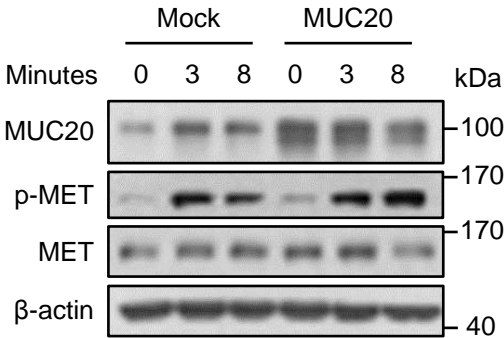

**B**

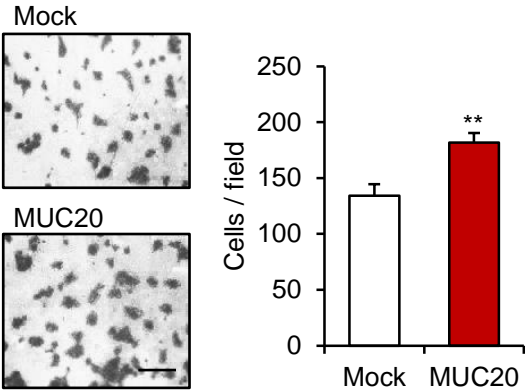

Supplement: Supplementary file 7 — Supplementary figure 7 [file 41388_2018_403_MOESM7_ESM.pdf]

# Supplementary figure S8

**A**

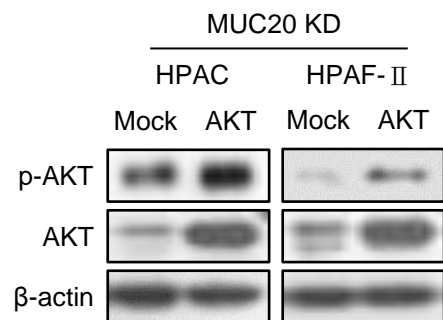

**B**

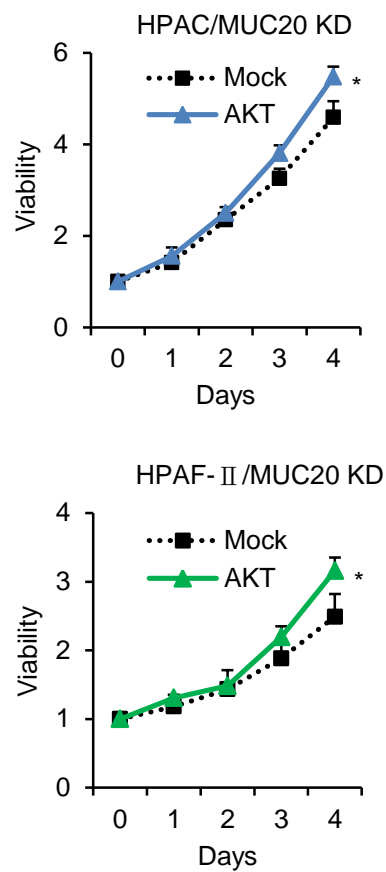

**C**

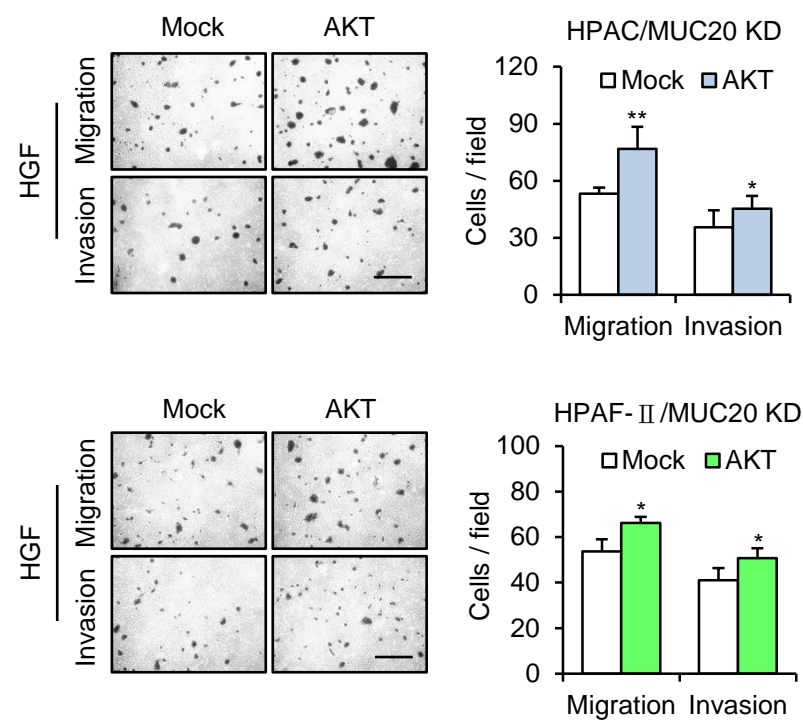

Supplement: Supplementary file 8 — Supplementary figure 8 [file 41388_2018_403_MOESM8_ESM.pdf]
