# Supplementary material for: Silencing of MUC20 suppresses the malignant character of pancreatic ductal adenocarcinoma cells through inhibition of the HGF/MET pathway
Source: Oncogene. 2018 Jul 11;37(46):6041–53. doi: 10.1038/s41388-018-0403-0 (PMC6237765; doi:10.1038/s41388-018-0403-0)
Supplement: Supplementary file 9 — Supplementary figure 9 [file 41388_2018_403_MOESM9_ESM.docx]

**Supplementary information**

**Supplementary figure S1. MUC20 expression in the primary PDAC tissue.** Representative images of MUC20 expression in the primary PDAC tissue by immunohistochemical staining. Left, low magnification. Right, high magnification. Arrow heads indicate the apical surface of adenocarcinoma cells. Scale bars indicate 50 μm.

**Supplementary figure S2. MUC20 is induced by serum deprivation.** HPAC and HPAF-II cells were cultured in media containing different concentrations of FBS, as indicated, for 24 hours. MUC20 expression levels were analysed by Western blotting. β-actin was used as an internal control.

**Supplementary figure S3. The JNK signalling pathway is involved in MUC20 overexpression induced by serum deprivation in HPAC and HPAF-II cells.** (**A**) Real-time RT-PCR revealed that *MUC20* mRNA levels were upregulated by serum deprivation. β-actin was used as an internal control. **, *P* < 0.01. (**B**) Western blots showing that serum deprivation increased MUC20 and p-JNK, but not p-p38 levels. (**C**) The JNK inhibitor SP600125 decreased p-JNK and MUC20 levels induced by serum deprivation. PDAC cells were pretreated with DMSO or SP600125 for 1 hour and then cultured in media containing 10% or 0% FBS for 24 hours.

**Supplementary figure S4. MUC20 knockdown does not significantly affect 1% FBS-triggered migration and invasion in HPAC and HPAF-II cells.** Transwell migration assay and Matrigel invasion assay were performed in 1% FBS / DMEM-F12 to analyse migration and invasion, respectively. Left, representative images of migrated and invaded cells. Right, statistics of cell migration and invasion. Scale bars indicate 1 mm.

**Supplementary figure S5. *HGF* mRNA is up-regulated by serum deprivation in PSCs.** Pancreatic stellate cells (PSCs) were cultured in DMEM-F12 containing 10% or 1% FBS for 24 hours. The mRNA expression of *HGF* was analysed by real-time RT-PCR. β-actin was used as an internal control. **, *P* < 0.01.

**Supplementary figure S6.** **MUC20 knockdown inhibited HGF-triggered phosphorylation of MET and AKT but not ERK and NF-kB in HPAC and HPAF-II cells.** (**A**) MUC20 knockdown inhibited p-MET and p-AKT in HPAF-II cells treated with 25 ng/ml HGF for different time periods, as indicated. (**B**) MUC20 knockdown did not affect the p-ERK and p-NF-kB in HPAC and HPAF-II cells treated with 25 ng/ml HGF.

**Supplementary figure S7.** **MUC20 overexpression increases p-MET and cell migration triggered by HGF in HPAF-II cells.** (**A**) MUC20 overexpression increased p-MET in HPAF-II cells treated with 25 ng/ml HGF for different time periods, as indicated. (**B**) MUC20 overexpression increased HGF-triggered migration in HPAF-II cells analysed by Transwell migration assays. Left, representative images of migrated cells. Right, statistics of cell migration. Scale bar indicates 1 mm. **, *P* < 0.01.

**Supplementary figure S8.** **AKT overexpression increased HGF-triggered viability, migration, and invasion in stable MUC20 knockdown cells.** (**A**) Stable MUC20 knockdown (KD) cells of HPAC and HPAF-II were transiently transfected with vector (Mock) or AKT plasmid (AKT) and then analysed by Western blotting. (**B**) MTT assays showed that AKT overexpression increased viability of HPAC and HPAF-II cells with stable MUC20 knockdown. *, *P* < 0.05. (**C**) AKT overexpression enhanced HGF-triggered migration and invasion in HPAC and HPAF-II cells with MUC20 knockdown analysed using Transwell migration and Matrigel invasion assay, respectively. Scale bars indicate 1 mm.*, *P* < 0.05. **, *P* < 0.01.

**Supplementary figure S9. MUC20 levels in MUC20 knockdown cells are also lower than control cells under serum deprivation conditions.** MUC20 expression levels were lower in MUC20 knockdown cells compared with control cells at 10% FBS or without FBS analysed by Western blotting. HPAC and HPAF-II cells were transfected with control (si-Control) or different MUC20 siRNAs (si-MUC20-1, -2, -3, and -4) and then cultured in media containing different concentrations of FBS, as indicated, for 24 hours. MUC20 was decreased in cells transfected with all siRNAs except for si-MUC20-4. β-actin was used as an internal control.
