# Supplementary material for: Silencing of MUC20 suppresses the malignant character of pancreatic ductal adenocarcinoma cells through inhibition of the HGF/MET pathway
Source: Oncogene. 2018 Jul 11;37(46):6041–53. doi: 10.1038/s41388-018-0403-0 (PMC6237765; doi:10.1038/s41388-018-0403-0)
Supplement: Supplementary file 10 — Supplementary Figure Legends [file 41388_2018_403_MOESM10_ESM.pdf]

Figure for review only

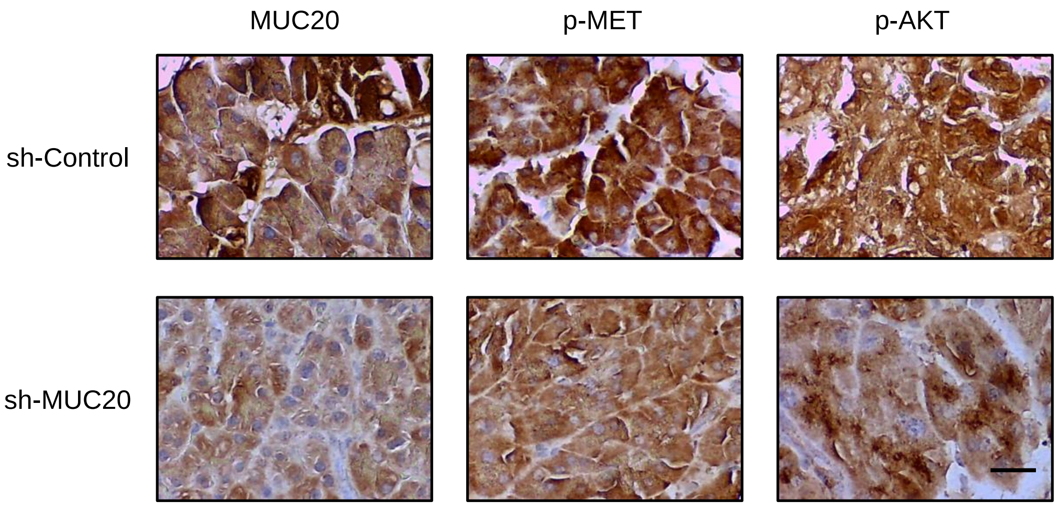

Figure for review only. Representative images of IHC staining of MUC20, p-MET, or p-AKT in orthotopic xenografts with control (sh-Control) or MUC20 knockdown (sh-MUC20). Scale bar indicates 20 µm.
